# Accuracy of AI Tools in the Diagnosis of Benign, Potentially Malignant and Malignant Oral Lesions: A Pilot Study

**DOI:** 10.3390/jcm15072638

**Published:** 2026-03-30

**Authors:** Luis Monteiro, Juliana Lima, Luís Silva, Caren Kaur Jauhal, Aaya Shamekh, Vlaho Brailo, Danica Vidović Juras, Ali Alqarni, Khalid Al-Johani, Sara Ferreira, Filomena Salazar, Molly Harte, Rui Albuquerque

**Affiliations:** 1 UNIPRO—Oral Pathology and Rehabilitation Research Unit, University Institute of Health Sciences (IUCS-CESPU), 4585-116 Gandra, Portugal; 2 Department of Oral Medicine, Guy’s and St Thomas NHS Foundation Trust, London SE1 9RT, UK; 3 Clinic for Dentistry, School of Dental Medicine, University Clinical Hospital Centre, University of Zagreb, 10000 Zagreb, Croatia; 4 Department of Oral & Maxillofacial Surgery and Diagnostic Sciences, Faculty of Dentistry, Taif University, Taif 21944, Saudi Arabia; 5 Diagnostic Sciences Department, Faculty of Dentistry, King Abdulaziz University, Jeddah 21589, Saudi Arabia; 6 Faculty of Dentistry, Oral and Craniofacial Sciences, King’s College London, London SE1 9RT, UK

**Keywords:** artificial intelligence, oral potentially malignant disorders, oral cancer, mouth mucosa, computer-assisted diagnosis, clinical decision-making, diagnosis

## Abstract

**Background:** Artificial intelligence (AI) is expected to play an increasingly important role in medicine and dentistry. While its diagnostic potential has been tested in various medical fields, limited research exists on its applications within oral medicine diagnoses using clinical images. **Objective:** This pilot study aimed to evaluate the diagnostic accuracy of ChatGPT, Gemini, and Copilot in identifying benign, potentially malignant, and malignant oral lesions. **Methods:** A cross-sectional study was conducted using clinical images from three categories: benign oromucosal conditions, oral potentially malignant disorders, and malignant oral lesions. **Results:** ChatGPT evaluated all images and consistently outperformed Copilot—and in some cases Gemini—across multiple diagnostic questions, with statistically significant advantages particularly in the cancer subgroup. Copilot showed the weakest performance, with high rates of missing evaluations and significantly lower proportions of correct responses in several analyses. Across both full-dataset and adjusted analyses, ChatGPT demonstrated the highest diagnostic performance overall. Diagnostic accuracy metrics for malignancy suspicion was similar for ChatGPT and Gemini. Several limitations such as sample size, lack of reproducibility testing and inability of some AI models to process images must be taken into account when interpreting the results. **Conclusions:** AI tools show promise but cannot yet replace clinical expertise. Further research and development are needed to improve the accuracy and applicability of AI diagnostic tools.

## 1. Introduction

Over the past two decades, artificial intelligence (AI) tools have demonstrated impressive capabilities in healthcare settings, particularly in medical diagnostics [1,2,3]. AI does offer some advantages over human intelligence (HI), with better ability to multi-task and better retention of information. However, HI continues to surpass AI in areas such as adaptability, creativity, emotional understanding and abstract reasoning [4]. Nonetheless, when AI systems produce accurate and reliable results, collaboration between AI and HI may be mutually beneficial [5]. Numerous studies have documented the successful application of machine learning-based AI systems in dental diagnoses including coronal and apical odontogenic pathology [6,7], Sjögren’s disease [8] and oral cancers [9]. These systems are typically custom-designed to detect a specific pathology, and are trained and validated on large, condition-specific and population-representative datasets to ensure accuracy and validity. In this manuscript, we focus on general-purpose multimodal LLMs (large language models), which are not trained or validated as dedicated diagnostic imaging systems, and we evaluate their performance only as potential clinical decision-support adjuncts.

In contrast to AI systems purposely designed for medical diagnostic use, a number of open access AI systems exist that may have a role in the healthcare setting with reduced associated cost and training requirements. ChatGPT (OpenAI, San Francisco, CA, USA) is a large language model (LLM) developed by OpenAI, designed to understand and generate human-like text in response to user input [10]. Beyond generating coherent responses, ChatGPT can perform a wide range of tasks, including answering questions, engaging in conversation and generating code. GPT-3.5 and GPT-4, the third and fourth iterations of ChatGPT, have multimodal capabilities and can process various input formats, including text and images [11]. This capability holds significant potential for applications in medical diagnostics. Copilot (Microsoft Corporation, Redmond, WA, USA), developed by Microsoft using the GPT-4 architecture, is another generative AI system with similar capabilities [12]. Gemini Pro (Google LLC, Mountain View, CA, USA) is Google’s premium AI software, part of the broader Gemini family, designed to compete with ChatGPT and other advanced systems. It supports natural language processing (NLP), computer vision, and multimodal input processing [13]. ChatGPT, Copilot, and Gemini are all pre-trained open access AI systems that interact intelligently with users, leveraging neural networks to process natural language and generate coherent, contextually appropriate responses. Their capabilities extend to pattern recognition and predictive modelling, including image analysis, which could be applied to the identification of abnormalities in medical images [14].

The potential application of ChatGPT in oral medicine has been explored in recent literature. For instance, de Souza et al. [15] suggested several benefits of ChatGPT in oral medicine, including providing both patients and clinicians with clinical information and assisting with clinical documentation. Diniz-Freitas et al. [16] assessed ChatGPT’s knowledge of oral potentially malignant disorders (OPMDs) using a series of guideline-based questions. Whilst ChatGPT demonstrated a moderate knowledge base, limitations were observed. Notably, these studies have found that ambiguities in the input, such as unclear or conflicting guidelines, often lead to inaccuracies in the output [16]. Uranbey et al. [17] evaluated the diagnostic accuracy of ChatGPT-3.5 using textual clinical vignettes and found that its performance was comparable to that of human clinicians. Kaygisiz and Teke [18] evaluated the diagnostic accuracy of ChatGPT-4 using imagined text-only patient scenarios with moderate accuracy. However, to date, no studies have assessed and compared the diagnostic capabilities of ChatGPT, Gemini, or Copilot in oral medicine using visual input.

The aim of this pilot study was to evaluate the diagnostic accuracy of three AI platforms, ChatGPT (version GTP-4o), Gemini (version Gemini Pro), and Copilot (Microsoft Copilot Pro), for potentially malignant and malignant oral lesions from clinical images. The secondary aim was to assess the three platforms’ ability to recommend appropriate diagnostic investigations.

## 2. Materials and Methods

Ethical approval for this study was granted by the Ethics Committee of IUCS—CESPU (Decision No. 07/CE-IUCS/2025) and the study was conducted in accordance with the Declaration of Helsinki. The study was carried out at the Dental Clinic of the University Clinic of the Instituto Universitário de Ciências da Saúde (IUCS), CESPU, Gandra, Portugal. Informed consent was obtained from all individuals whose clinical images were used, and all data were anonymized. The images were used exclusively for academic and research purposes.

**Figure 1:**
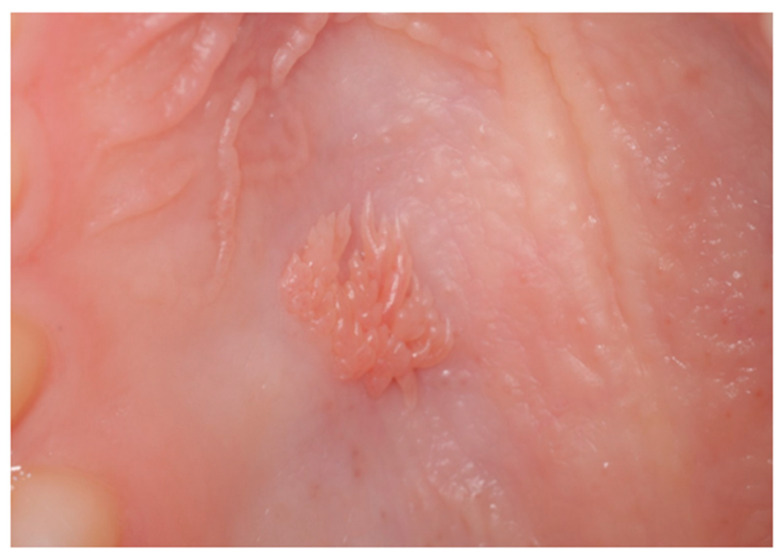
Squamous cell papilloma (benign) on the hard palate.

**Figure 2:**
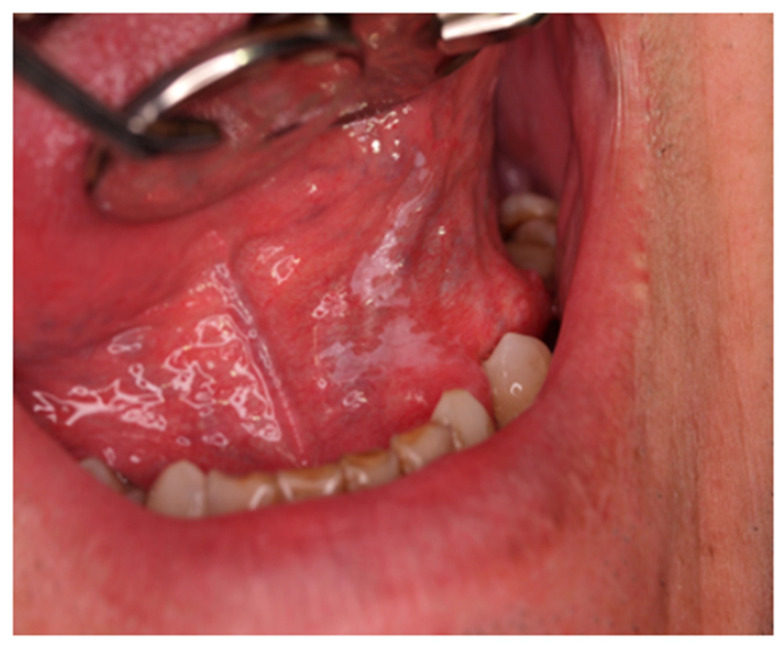
Leukoplakia (potentially malignant) on the floor of the mouth.

**Figure 3:**
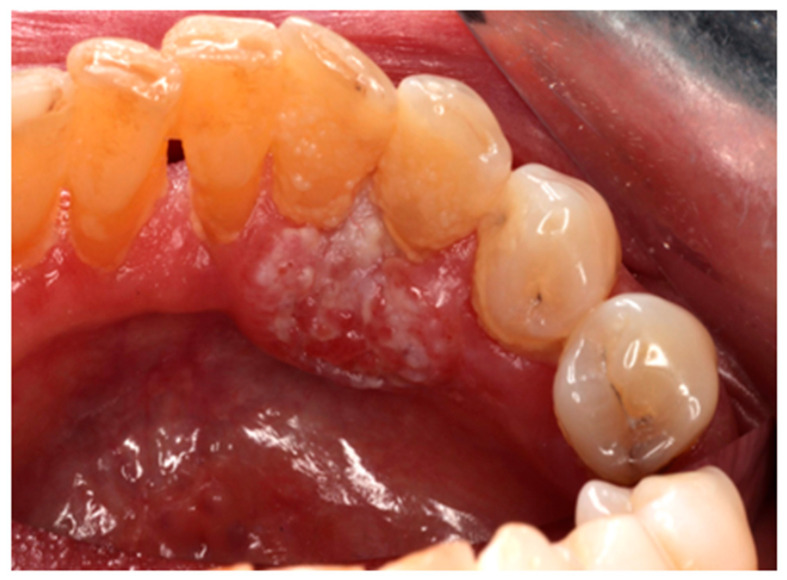
Squamous cell carcinoma (malignant) on the lower lingual gingivae.

The clinical images used in this pilot study were obtained using a Canon EOS DSLR 90D^®^ (Canon Inc., Tokyo, Japan), 50 mm macro lens, using a ring flash, under the same lighting conditions, and focusing the oral lesions. The images were separately evaluated and assigned clinical diagnoses by two oral medicine specialists, with 100% diagnostic agreement reached prior to input into the AI programs. When indicated, the final diagnosis was reached according to complementary investigations such as histopathology examination. Examples of oral lesions included in the study are squamous cell papillomata, leukoplakias and squamous cell carcinomas (Figure 1, Figure 2 and Figure 3).

The dataset comprised 30 JPEG clinical images selected from a pre-existing database of patients from the Medicine and Oral Surgery Department, IUCS—CESPU (Supplementary Table S1). The sample size was determined for this pilot study based on estimation precision of overall diagnostic accuracy rather than formal hypothesis testing. Using the standard formula for estimating a proportion,

n = (Z^2^ × p(1 − p))/MOE^2^

where Z = 1.96 (95% confidence level), p = 0.5 (maximizing variance and therefore producing the most conservative sample size), and margin of error (MOE) = 0.18, the required sample size was approximately 29.64. A proportion of 0.5 was chosen to represent the worst-case scenario for variance when the true diagnostic accuracy is unknown. This calculation yields a precision of ±18% around the estimated overall accuracy, which is acceptable for exploratory pilot investigations. The final sample size was therefore set at 30 images.

To prevent class imbalance from artificially influencing overall accuracy estimates, the images were equally distributed across three diagnostic categories: 10 benign lesions (“Lumps”), 10 oral potentially malignant disorders (“OPMDs”), and 10 malignant lesions (“Oral Cancer”) (Supplementary Table S1). Unequal class representation can bias accuracy metrics by overrepresenting the most prevalent class, thereby inflating apparent model performance. Equal allocation ensured balanced evaluation of model performance across clinically distinct diagnostic groups.

The images were simultaneously submitted to the three different versions of AI models: ChatGPT (version GTP-4o, OpenAI, San Francisco, CA, USA), Gemini (version Gemini Pro, Google LLC, Mountain View, CA, USA), and Copilot (Microsoft Copilot Pro, Microsoft Corporation, Redmond, WA, USA). Each model was provided with the same image set, with each image submitted in isolation. The order of image submission was randomized. No additional clinical data were provided beyond the image itself. The analysis on the three AI models was performed on 24 June 2025.

For each image, the AI models were asked to answer a standardized set of four questions. No additional information was given, and no other prompts were used.

1. What is the most probable diagnosis of the observed lesion?
2. What are the other possible differential diagnoses? (Please indicate in order of relevance)
3. Do you think the lesion is suspicious for oral cancer? (Please answer Yes or No)
4. What complementary exams would you suggest for lesion investigation? (Please indicate in order of relevance)

The responses from each model were recorded and organized.

An analysis grid was developed using objective, pre-defined criteria to assess the accuracy of each response. Each criterion was scored using a binary system (0 = incorrect or no response; 1 = correct response). The score was based upon the pre-defined clinical diagnoses.

The four assessment criteria were:

1. Most likely diagnosis—1 point if the model’s answer matched the gold standard.
2. Inclusion of the correct diagnosis in the differential list—1 point if the correct diagnosis appeared in the suggested differential diagnosis list. There was no limit placed on the number of responses which could be provided by the AI model.
3. Assessment of suspected oral cancer—1 point if the model’s response correctly identified (or excluded) malignancy.
4. Suggestion of an appropriate complementary exam—1 point if the model suggested the most clinically relevant investigation. There was no limit placed on the number of responses which could be provided by the AI model.

Given that some AI models were unable to process all images, two analytical approaches were undertaken to assess the robustness of results in relation to the missing data.

In the primary (conservative) analysis, unprocessed images were treated as incorrect responses (coded as 0), reflecting real-world clinical usability in which failure to analyze an image presents a diagnostic limitation.

Diagnostic accuracy analysis was subsequently performed using a complete-case approach, in which unprocessed images were excluded from the sample and diagnostic accuracy was recalculated using only successfully analyzed images. This approach allowed evaluation of diagnostic reasoning performance independent of technical processing failures.

Comparison of these two analytical scenarios enabled assessment of the extent to which observed differences between platforms were influenced by missing data handling assumptions.

Diagnostic accuracy metrics (sensitivity, specificity, accuracy, PPV, and NPV) were calculated for malignancy suspicion (Q3) using a 2 times 2 contingency table (including true positive [TP], false positive [FP], true negative [TN], false negative [FN]) for ChatGPT, Gemini, and Copilot and considering both analytic scenarios based on how unprocessed photographs were treated. The following metrics and their corresponding 95% confidence intervals (95% CIs) were calculated:

- Sensitivity: The model’s ability to correctly identify positive cases (malignant).

Sensitivity = TP/(TP + FN)

- Specificity: The model’s ability to correctly identify negative cases (non-malignant cases).

Specificity = TN/(TN + FP)

- Accuracy: The overall proportion of correct diagnoses among all processed cases.

Accuracy = (TP + TN)/(TP + TN + FP + FN)

- Positive Predictive Value (PPV): The probability that a case identified as “positive” by the AI is truly positive.

PPV = TP/(TP + FP}

- Negative Predictive Value (NPV): The probability that a case identified as “negative” by the AI is truly negative.

NPV = TN/(TN + FN)

Statistical differences in diagnostic accuracy metrics’ proportions across the three models were assessed using Fisher’s exact test (due to counts < 5 in some cells). For significant global results, post hoc pairwise comparisons were performed using Fisher’s exact test with a significance threshold of *p* < 0.05.

The primary outcome was correct identification of the most probable diagnosis (Q1). Differential diagnosis (Q2) was evaluated only when the primary diagnosis did not match the reference standard. This hierarchical design was intended to approximate clinical reasoning, whereby alternative diagnoses are considered when the initial diagnosis is incorrect. However, this approach introduces conditional dependency between Q1 and Q2 outcomes and therefore precludes independent statistical interpretation of Q2 as a standalone diagnostic metric. For interpretative purposes, Q1 and Q2 were combined into a composite measure termed hierarchical diagnostic capture, defined as correct identification either as the primary diagnosis or, if incorrect, within the differential list.

Data were coded and saved in an Excel worksheet. All data were categorical and expressed as absolute frequencies and proportions. Each outcome variable was binary (correct response = 1; incorrect = 0). Statistical analyses were performed using IBM SPSS Statistics for Windows (Version 23.0; IBM Corp., Armonk, NY, USA). Comparisons of proportions of correct responses between the three AI models were conducted using the Chi-square test of independence. When expected cell counts were less than five, Fisher’s exact test was applied as appropriate. Cramer’s V was calculated as the measurement of the effect size (0.07–0.21—small effect; 0.21–0.35—moderate effect; >0.35—large effect).

Sensitivity and specificity for malignancy suspicion (Q3) were calculated as exploratory performance indicators. Given the limited number of malignant cases (n = 10), these estimates are reported with 95% confidence intervals to reflect their imprecision and should be interpreted as preliminary. *p*-values lower than *p* < 0.05 were considered statistically significant.

## 3. Results

Not all images were successfully evaluated by every AI model. Gemini was unable to process three images, corresponding to 10% of the dataset. Copilot failed to evaluate 12 images, accounting for 40% of the total. In contrast, ChatGPT successfully evaluated all 30 images. The responses to all questions are presented in Supplementary Tables S2A–C, S3A–C and S4A–C.

**Figure 4:**
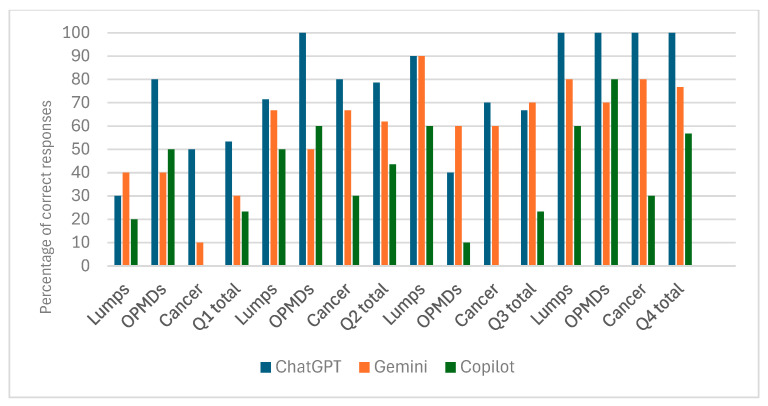
Bar chart illustrating data in Table 1.

Table 1 (and Figure 4) presents the results of diagnostic accuracy testing with all photographs, where missing responses were counted as incorrect answers. For question 1, where the AI systems were asked to provide the probable diagnosis, a significant difference in the proportion of correct responses between different AI models was observed (*p* = 0.039, V = 0.178, indicating a small effect). However, post hoc testing did not detect a significant difference, which is likely due to the small sample size. In the subgroup analysis, a significant difference in the proportion of correct responses between different AI models was found only in the “Cancer” category (*p* = 0.013, V = 0.540, indicating a strong effect). Post hoc testing detected that ChatGPT had a significantly higher proportion of correct responses compared to Copilot (5; 50% vs. 0; *p* = 0.0037).

For question 2, where the AI models were asked to provide a differential diagnosis, no significant difference in the proportion of correct responses between different AI models was found (*p* = 0.102, V = 0.281, indicating a moderate effect). For question 1 and question 2 combined, a significant difference in the proportion of correct responses between different AI models was observed (*p* = 0.014, V = 0.308, indicating a moderate effect). Post hoc testing detected that ChatGPT had a significantly higher proportion of correct responses compared to Copilot (27; 90% vs. 17; 56.7%; *p* = 0.0124). In the subgroup analysis, a significant difference in the proportion of correct responses between different AI models was found only in the “Cancer” category (*p* = 0.018, V = 0.518, indicating a strong effect). Post hoc testing detected that ChatGPT had a significantly higher proportion of correct responses compared to Copilot (*p* = 0.0069).

For question 3, which asked whether the lesion was suspicious for cancer, a significant difference in the proportion of correct responses between different AI models was observed (*p* < 0.0001, V = 0.426, indicating a strong effect). Post hoc testing detected that both ChatGPT and Gemini had a significantly higher proportion of correct responses when compared with Copilot (20; 66.7% and 21; 70% vs. 7; 23.3%; *p* = 0.00006). In the subgroup analysis, a significant difference in the proportion of correct responses between different AI models was found only in the “Cancer” category (*p* = 0.0002, V = 0.624, indicating a strong effect). Post hoc testing detected that both ChatGPT and Gemini had a significantly higher proportion of correct responses when compared with Copilot (7; 70% and 6; 60% vs. 0; *p* = 0.00067).

For question 4, which asked which complementary investigations would be suggested to aid diagnosis, a significant difference in the proportion of correct responses between different AI models was observed (*p* < 0.0001, V = 0.426, indicating a strong effect). Post hoc testing detected that ChatGPT had a significantly higher proportion of correct responses compared to both Gemini and Copilot (30; 100% vs. 23; 76.7% and 17; 56.7%; *p* = 0.0003 and *p* = 0.0007). In the subgroup analysis, a significant difference in the proportion of correct responses between different AI models was found only in the “Cancer” category (*p* = 0.002, V = 0.642, indicating a strong effect). Post hoc testing detected that ChatGPT had a significantly higher proportion of correct responses compared to Copilot (10; 100% vs. 3; 30%; *p* = 0.00067).

**Figure 5:**
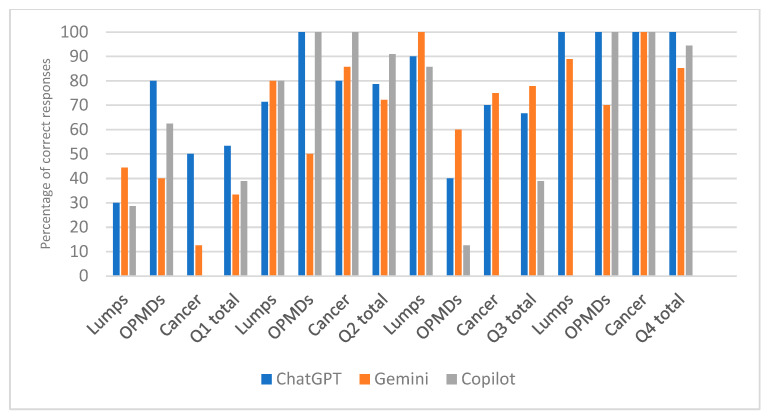
Bar chart illustrating data in Table 2.

Table 2 (and Figure 5) presents the results of diagnostic accuracy testing where unprocessed photographs were removed from the analysis. ChatGPT and Gemini managed to process significantly more photographs compared to Copilot (30; 100% and 27; 90% vs. 18; 60%; *p* < 0.0001, V = 0.465, indicating a strong effect). A subgroup analysis detected that ChatGPT and Gemini managed to process significantly more oral cancer photographs compared to Copilot (10; 100% and 8; 80% vs. 3; 30%; *p* = 0.002, V = 0.642, indicating a strong effect).

For question 1, no significant difference in the proportion of correct responses was observed between different AI models. A subgroup analysis did not detect a significant difference in the percentage of correct responses between different AI models for any of the subgroups.

For question 2, no significant difference in the proportion of correct responses was observed between different AI models. A subgroup analysis did not detect a significant difference in the percentage of correct responses between different AI models for any of the subgroups.

For questions 1 and 2 combined, no significant difference in the proportion of correct responses was observed between different AI models. A significant difference in the percentage of correct responses between different AI models was observed in the OPMD subgroup (*p* = 0.049, V = 0.465, indicating a strong effect). However, post hoc pairwise comparisons did not detect a significant difference due to the small sample size.

For question 3, a significant difference in the proportion of correct responses was found between different AI models (*p* = 0.027, V = 0.311, indicating a moderate effect). Post hoc testing detected that Gemini had significantly more correct responses when compared with Copilot (21; 77.8% vs. 7; 38.9%; *p* = 0.027). A subgroup analysis did not detect a significant difference in the percentage of correct responses between different AI models for any of the subgroups.

For question 4, no significant difference in the proportion of correct responses was observed between different AI models. A significant difference in the percentage of correct responses between different AI models was observed in the OPMD subgroup (*p* = 0.049, V = 0.465, indicating a strong effect). However, post hoc pairwise comparisons did not detect significant differences due to the small sample size.

Table 3 presents diagnostic accuracy metrics analysis of AI models in detecting oral cancer (Q3) using both approaches (treating unprocessed images as incorrect responses and excluding unprocessed images from the sample). The overall sensitivity of all AI models in detecting oral cancer was 43.3% (25.5–62.6%) when unprocessed images were treated as incorrect responses and 61.90% (38.4–81.9%) when unprocessed images were excluded from the sample. ChatGPT achieved the highest sensitivity (70.00%) and a specificity of 65.00%, resulting in a consistent overall accuracy of 66.67% across both analytical scenarios. Gemini achieved an accuracy of 70.00%, a sensitivity of 60.00%, and the highest specificity at 75.00% in the “unprocessed as 0” scenario. When unprocessed images were excluded, Gemini’s performance increased to an accuracy of 77.78%, a sensitivity of 75.00%, and a specificity of 78.95%. Copilot recorded the lowest values, with 0% sensitivity, 35.00% specificity, and an accuracy of only 23.33%. These low values persisted even when excluding unprocessed images, yielding a specificity of 46.67%, an accuracy of 38.89%, and a sensitivity of 0%.

As detailed in Supplementary Table S5, global statistical analysis confirmed significant differences between the platforms in considering the “unprocessed photographs as incorrect” scenario across all metrics (*p* < 0.05). Pairwise comparisons using Fisher’s exact test indicated that both ChatGPT and Gemini were significantly superior to Copilot in terms of accuracy, sensitivity, and Positive Predictive Value (PPV). Notably, Gemini maintained a statistically significant accuracy advantage over Copilot (*p* = 0.013) even when image rejections were excluded. No statistically significant differences were observed between ChatGPT and Gemini for any metric or scenario (*p* > 0.05).

## 4. Discussion

This pilot study demonstrates considerable variability in diagnostic performance among ChatGPT, Gemini, and Copilot when applied to clinical images of oral lesions. Although the models frequently included the correct diagnosis within their differential lists, they often failed to identify it as the most probable diagnosis. This limitation is clinically meaningful, as accurate diagnostic prioritization is central to safe decision-making. Copilot frequently included the correct diagnosis within its differential lists despite lower accuracy in identifying it as the most probable diagnosis. However, this pattern should not be interpreted as evidence of high diagnostic accuracy. The platform demonstrated a substantial rate of unprocessed images, particularly within the cancer subgroup, and showed lower accuracy in correctly identifying lesions as suspicious for malignancy (Q3). Therefore, its broader differential listings might likely reflect non-specific response generation rather than consistent detection of truly suspicious lesions. ChatGPT showed accuracy for provision of differential diagnoses in the OPMD section. This aligns with prior research indicating that LLMs, despite limitations in ranking the correct diagnosis first, often recognize the correct condition as a valid possibility [19]. The present study showed their current limitations, especially in high-risk or malignant cases, reinforcing the necessity of clinician oversight and further domain-specific development. A clear performance difference emerged in assessing suspicion of malignancy. When missing responses were included, ChatGPT and Gemini were significantly more accurate than Copilot, driven partly by superior performance in oral cancer cases.

However, these differences were partly attributable to Copilot’s higher rate of image rejection (40%), especially for malignant lesions. Therefore, part of the observed superiority of ChatGPT and Gemini reflects differences in image processing capability rather than purely diagnostic reasoning performance. This introduces a potential bias if interpreted solely as diagnostic superiority. The accuracy analysis excluding unprocessed images demonstrated attenuation of several statistically significant findings, indicating that differential moderation and image processing restrictions substantially contributed to observed performance differences. Therefore, comparisons between platforms must be interpreted cautiously, distinguishing between technical usability and diagnostic reasoning accuracy.

ChatGPT showed high accuracy in recommending appropriate complementary diagnostic investigations such as biopsies with 100% accuracy in all groups, outperforming both Gemini and Copilot. ChatGPT performed better when naming possible differential diagnoses than when asked for the most probable diagnosis of the observed lesion. This reinforces its value as an assistive tool rather than providing primary diagnosis. These results support the use of LLMs as adjuncts, rather than replacements, for clinical reasoning.

Because general-purpose multimodal models can often include the correct condition in their differential diagnoses list, they can be positioned early in the decision tree to support hypothesis generation and initial triage, rather than making definitive diagnoses. Their frequent failure to rank the correct diagnosis first means clinicians must retain control over diagnostic prioritization. As a result, the decision tree shifts towards using AI as an adjunct for differential diagnosis while keeping final clinical judgment and management firmly clinician-led.

Excluding unprocessed images substantially modified performance patterns. Gemini and ChatGPT processed significantly more images than Copilot, the latter failing to analyze 40% of the dataset—particularly cancer cases—due to system moderation restrictions. Once these cases were removed, differences in diagnostic accuracy for the first and second questions were no longer statistically significant. For the third question, Gemini retained a significant advantage over Copilot, though subgroup analyses no longer reached significance. No significant differences were detected for the fourth question after excluding unprocessed cases, despite ChatGPT maintaining numerically superior performance. These contrasting results highlight the methodological challenge posed by inconsistent image processing across platforms: analytic outcomes depend heavily on whether unprocessed images are considered diagnostic failures or removed entirely.

The current findings contrast sharply with multiple meta-analyses showing that imaging-based AI systems, particularly deep learning models, consistently achieve higher sensitivity (often >90%) in malignancy detection [17,20,21,22]. For example, a 2024 meta-analysis reported a pooled sensitivity of approximately 90%, specificity of 89%, and area under the ROC curve (AUC) of 0.94 for detecting oral cancer and OPMDs using dedicated image-trained AI models [21]. Similarly, a 2022 review reported diagnostic odds ratios above 60, with most systems achieving over 80% in both sensitivity and specificity when analyzing clinical or radiographic images [23]. ChatGPT’s consistently high accuracy in recommending appropriate investigations and Gemini’s relative strength in recognizing malignancy suspicion suggest that general-purpose LLMs can extract clinically relevant visual cues and provide meaningful reasoning support to an extent.

While this study provides valuable preliminary evidence on the diagnostic performance of multimodal AI systems in oral medicine, several methodological and technical limitations must be acknowledged when interpreting the results. These limitations align with broader concerns reported in recent oral-pathology chatbot evaluations, including variable accuracy, inconsistent outputs, and reliability/citation issues.

The relatively small sample size constitutes one of the principal limitations of this study. Only thirty clinical images were included, divided equally amongst the benign, potentially malignant and malignant lesion categories. Although this allows for structured comparison across diagnostic groups, the limited number of cases restricts statistical power and reduces the capacity to detect meaningful differences in model performance. A formal sample size calculation based on sensitivity and specificity was not performed, as reliable preliminary performance estimates for multimodal large language models in oral lesion image analysis were unavailable at the time of study design. Consequently, the study should be interpreted as exploratory and hypothesis-generating rather than as a definitive diagnostic validation. Larger, prospectively powered studies will be required to establish precise sensitivity and specificity estimates. We did, however, calculate the sensitivity and specificity for malignancy suspicion (Q3) in our sample as performance indicators (in diagnostic accuracy metric performance analysis). The limited sample size resulted in wide confidence intervals for sensitivity and specificity estimates. These findings should therefore be interpreted cautiously and considered hypothesis-generating. Larger, prospectively powered studies will be required to obtain precise and generalizable diagnostic performance estimates.

Furthermore, all images were sourced from a single academic institution in Portugal. Whilst this ensures methodological consistency and ethical control, it also limits the demographic diversity represented within the dataset. The sample may not capture the full clinical variability of oral lesions across different ethnicities, geographic regions, and healthcare settings. Consequently, the findings cannot be generalized to the wider population without caution. A larger and more diverse dataset would have enhanced the reliability of the accuracy metrics and allowed for more comprehensive subgroup analyses, such as comparisons by lesion location. The conditional evaluation of differential diagnoses represents a methodological limitation. Because Q2 was assessed only when Q1 was incorrect, differential performance metrics are inherently dependent on primary diagnostic accuracy and cannot be interpreted as independent measures of diagnostic breadth. The combined Q1 + Q2 metric therefore reflects hierarchical diagnostic capture rather than pure diagnostic accuracy. While this approach approximates certain aspects of clinical reasoning, it introduces interpretative complexity and may influence comparative findings. Future investigations should independently evaluate primary and differential diagnoses for all cases to enable clearer separation of diagnostic prioritization and recognition capacity.

The use of binary scoring represents a methodological simplification. Diagnostic reasoning is inherently multidimensional, and some responses that were classified as incorrect may have represented clinically reasonable alternatives or partial recognition of relevant pathology. Although inclusion of the correct diagnosis within differential lists was evaluated separately to mitigate this limitation, the binary scoring system does not fully capture all particularities of diagnostic prioritization or near-miss performance. Future investigations may benefit from more sophisticated scoring systems that better reflect the complexity of clinical reasoning while maintaining reproducibility. Development of such standardized tools for the assessment of multimodal AI diagnostic reasoning should be one of the methodological priorities in this emerging field.

In clinical practice, clinicians typically integrate multiple sources of data when making diagnostic decisions—not only the appearance of the lesion, but the clinical history, patient age, lesion location and medical history. No such supplementary information was provided to the AI platforms. In addition, each image was analyzed in isolation, preventing evaluation of lesion progression and comparison, leading to loss of clinically relevant diagnostic information. This approach, though necessary for study control, limits the ecological validity of the findings. This study adopted a cross-sectional design, with each AI model evaluated at a single time point. No repeated testing was performed to assess intra-model consistency or reproducibility of responses. Given that LLM outputs may vary depending on prompt phrasing and system updates, the results might differ if the same analysis were repeated. The absence of a reproducibility assessment therefore limits the strength of the conclusions drawn regarding model reliability.

A significant procedural limitation was the inability of some AI platforms to process all images. These failures were typically due to system restrictions, image rejection, or content moderation protocols that classified certain lesion images as “sensitive”. Such inconsistencies limit comparative validity between systems and highlight the current instability of multimodal AI tools. These system-level moderation policies restrict diagnostic capacity and complicate direct comparison. Furthermore, the cross-sectional design did not allow assessment of reproducibility or temporal consistency, which are important considerations given the dynamic nature of LLM outputs.

Addressing these limitations will require larger, multi-institutional datasets incorporating a greater range of lesion types, ethnic backgrounds and image qualities. Integration of patient metadata, such as clinical history and risk factors, could substantially enhance diagnostic contextualization. Evaluation under controlled, standardized conditions using repeated testing will also be essential to determine reliability and reproducibility.

## 5. Conclusions

This pilot study comparing general-purpose LLMs adds to the growing body of evidence supporting the cautious and supervised use of AI in clinical medicine. However, its findings should be interpreted with caution. The methodology primarily evaluates performance on a limited number of isolated images rather than within real clinical settings, reflecting basic visual pattern recognition rather than comprehensive diagnostic reasoning.

Whilst AI systems like ChatGPT, Gemini, and Copilot show potential as supportive tools in oral diagnostics, they currently fall short of being dependable diagnostic agents. None of the models evaluated in this study are yet suitable to replace clinical judgment, and the risks associated with incorrect diagnoses remain significant. The evaluated systems are general-purpose models whose training likely includes limited examples of oral mucosal pathology. Their performance reflects broad reasoning ability rather than specialized diagnostic expertise.

The diagnostic limitations observed, especially the frequent failure to correctly identify malignant lesions, highlight that the included AI systems cannot yet be considered safe for unsupervised clinical application. The potential for misdiagnosis carries significant implications for patient safety. Over-reliance on unsupervised AI could lead to missed or delayed diagnoses, raising significant concerns about patient safety [24]. These models should be viewed strictly as clinical decision-support tools, not autonomous diagnostic systems. Excluding unprocessed images substantially modified performance patterns. The inability of AI models, particularly Copilot, to process certain images can significantly limit their use. Furthermore, issues such as hallucinated references, lack of transparency in decision-making, and training data bias must be addressed before these technologies can be safely integrated into the clinical environment [17].

To advance the clinical applicability of such systems, future research should incorporate larger, multi-institutional datasets with greater demographic and lesion diversity, integrate clinical metadata and compare general-purpose LLMs with domain-trained medical models. Rigorous reproducibility testing and transparency regarding model decision-making will be essential before safe integration into clinical workflows can be considered.

## Figures and Tables

**Table 1:** Diagnostic accuracy testing with unprocessed images included.

Group	ChatGPT n; % of Correct Responses (95% CI)	Gemini n; % of Correct Responses (95% CI)	Copilot n; % of Correct Responses (95% CI)	*p*-Value	Cramer’s V	Post Hoc Bonferroni Correction
Lumps	3; 30% (6.7–65.2%)	4; 40% (12.2–73.8%)	2; 20% (2.5–55.6%)	0.621	0.178	
OPMDs	8; 80% (44.4–97.5%)	4; 40% (12.2–73.8%)	5; 50% (18.7–81.3%)	0.171	0.343	
Cancer	5; 50% (18.7–81.3%)	1; 10% (0.3–44.5%)	0(0–30.8%)	0.013 *	0.540	cGPT > Cop; 0.0037 *
Q1 total	16; 53.3% (34.3–71.7%)	9; 30% (14.7–49.4%)	7; 23.3% (9.9–42.3%)	0.039 *	0.269	Post hoc test did not detect significant difference
Lumps	5; 71.4% (44.4–97.5%)	4; 66.7% (22.3–95.7%)	4; 50% (15.7–84.3%)	0.668	0.196	
OPMDs	2; 100% (15.8–100%)	3; 50% (11.8–88.2%)	3;60% (14.7–94.7%)	0.451	0.350	
Cancer	4; 80% (28.4–99.5%)	6; 66.7% (29.9–92.5%)	3; 30% (6.7–65.2%)	0.119	0.421	
Q2 total	11; 78.6% (49.2–95.3%)	13; 61.9% (38.4–81.9%)	10: 43.5% (23.2–65.5%)	0.102	0.281	
Lumps Q1 + Q2	8; 80% (29.3–96.3%)	8; 80% (44.4–97.5%)	6; 60% (26.2–87.8%)	0.506	0.213	
OPMDs Q1 + Q2	10; 100% (69.2–100%)	7; 70% (34.8–93.3%)	8; 80% (29.3–96.3%)	0.186	0.335	
Cancer Q1 + Q2	9; 90% (55.5–99.7%)	7; 70% (34.8–93.3%)	3; 30% (6.7–65.2%)	0.018 *	0.518	cGPT > Cop; 0.0069 *
Q1 + Q2 total	27; 90% (73.5–97.9%)	22; 73.3% (54.1–87.7%)	17; 56.7% (37.4–74.5%)	0.014 *	0.308	cGPT > Cop; 0.0124 *
Lumps	9; 90% (55.5–99.7%)	9; 90% (55.5–99.7%)	6; 60% (26.2–87.8%)	0.153	0.354	
OPMDs	4; 40% (12.2–73.8%)	6; 60% (26.2–87.8%)	1; 10% (0.3–44.5%)	0.065	0.426	
Cancer	7; 70% (34.8–93.3%)	6; 60% (26.2–87.8%)	0% (0–30.8%)	0.002 *	0.624	cGPT > Cop; 0.00067 * Gem > Cop; 0.00067 *
Q3 total	20; 66.7% (47.2–82.7%)	21; 70% (50–6–85.3%)	7; 23.3% (9.9–42.3%)	0.0003 *	0.426	cGPT > Cop; 0.00006 * Gem > Cop; 0.00006 *
Lumps	10; 100% (69.2–100%)	8; 80% (44.4–97.5%)	6; 60% (26.2%87.8%)	0.082	0.408	
OPMDs	10; 100% (69.2–100%)	7; 70% (34.8–93.3%)	8; 80% (44.4–97.5%)	0.186	0.335	
Cancer	10; 100% (69.2–100%)	8; 80% (44.4–97.5%)	3; 30% (6.7–65.2%)	0.002 *	0.642	cGPT > Cop; 0.00067 *
Q4 total	30; 100% (88.4&–100%)	23; 76.7% (57.7–90.1%)	17; 56.7% (37.4–47.5%)	0.0003 *	0.426	cGPT > Gem; 0.0003 * cGPT > Cop; 0.0007 *

* *p* < 0.05; legend: cGPT—ChatGPT, Gem—Gemini, Cop—Copilot.

**Table 2:** Diagnostic accuracy testing with unprocessed images removed.

**Unprocessed Photographs**						
	**ChatGPT** **n; %**	**Gemini** **n; %**	**Copilot** **n; %**	***p*** **-Value**	**Cramer’s V**	**Post Hoc** **Bonferroni** **Correction**
Total	0	3; 10%	12; 40%	0.00086 *	0.456	cGPT > Cop; 0.00003 * Gem > Cop; 0.00270 *
Lumps	0	1; 10%	3; 30%	0.133	0.367	
OPMDs	0	0	2; 20%	0.117	0.378	
Cancer	0	2; 20%	7; 70%	0.002 *	0.642	cGPT > Cop; 0.00067 * Gem > Cop; 0.01242 *
**Diagnostic Accuracy Testing**						
GROUP	**ChatGPT** n; % of correct responses (95% CI)	**Gemini** n; % of correct responses (95% CI)	**Copilot** n; % of correct responses (95% CI)	***p*** **-Value**	**Cramer’s V**	**Post Hoc** **Bonferroni** **Correction**
Lumps	3; 30% (6.7–65.2%)	4; 44.4% (13.7–78.8%)	2; 28.6% (3.7–71%)	0.744	0.151	
OPMDs	8; 80% (44.4–97.5%)	4; 40% (12.2–73.8%)	5; 62.5% (24.5–91.5%)	0.186	0.347	
Cancer	5; 50% (18.7–81.3%)	1;12.5% (0.3–52.7%)	0(0–70.8%)	0.107	0.461	
Q1 total	16; 53.3% (34.3–71.7%)	9; 33.3% (16.5–54%)	7; 38.9% (17.3–64.3%)	0.292	0.181	
Lumps	5; 71.4% (29–96.3%)	4; 80% (28.4–99.5%)	4; 80% (28.4–99.5%)	0.919	0.099	
OPMDs	2; 100% (15.8–100%)	3; 50% (11.8–88.2%)	3; 100% (29.2–100%)	0.179	0.559	
Cancer	4; 80% (28.4–99.5%)	6; 87.5% (42.1–99.6%)	3; 100% (29.2–100%)	0.719	0.210	
Q2 total	11; 78.6% (49.2–95.3%)	13; 72.2% (46.5–90.3%)	10; 90.9% (58.7–99.8%)	0.486	0.183	
Lumps Q1 + Q2	8; 80% (44.4–97.5%)	8; 88.9% (51.8–99.7%)	5; 83.3% (35.9–99.6%)	0.869	0.106	
OPMDs Q1 + Q2	10; 100% (69.2–100%)	7; 70% (34.8–93.3%)	8; 100% (63.1–100%)	0.049 *	0.465	Post hoc test did not detect significant difference
Cancer Q1 + Q2	9; 90% (55.5–99.7%)	7; 87.5% (47.3–99.7%)	3; 100% (29.2–100%)	0.818	0.818	
Q1 + Q2 total	27; 90% (73.5–97.9%)	22; 81.5% (61.9–93.7%)	16; 94.1% (71.3–99.9%)	0.411	0.155	
Lumps	9; 90% (55.5–99.7%)	9; 100% (66.4–100%)	8; 85.7% (42.1–99.6%)	0.534	0.220	
OPMDs	4; 40% (12.2–73.8%)	6; 60% (26.2–87.8%)	1; 12.5% (0.3–52.7%)	0.122	0.388	
Cancer	7; 70% (34.8–93.3%)	6; 75% (34.9–96.8%)	0(0–70.8%)	0.057	0.523	
Q3 total	20; 66.7% (47.2–82.7%)	21; 77.8% (57.7–91.4%)	7; 38.9% (17.3–64.3%)	0.027 *	0.311	Gem > Cop; 0.01242
Lumps	10; 100% (69.2–100%)	8; 88.9% (51.8–99.7%)	6; 85.7% (42.1–99.6%)	0.494	0.233	
OPMDs	10; 100%	7; 70% (34.8–93.3%)	8; 100% (63.1–100%)	0.049 *	0.465	Post hoc test did not detect significant difference
Cancer	10; 100% (69.2–100%)	8; 100 (63.1–100%)	3; 100% (29.2–100%)	/	/	
Q4 total	30; 100% (88.4–100%)	23; 85.2% (66.3–95.8%)	17; 94.4% (72.7–99.9%)	0.080	0.260	

* *p* < 0.05; legend: cGPT, ChatGPT; Gem, Gemini; Cop, Copilot; CI, confidence interval.

**Table 3:** Diagnostic accuracy metric performance of AI models in detecting oral cancer.

Accuracy Metrics of the Results									
Metrics	“Unprocessed Photographs” Model	ChatGPT		Gemini		Copilot		All AI Systems	
		Correct Answer (n)	%	Correct Answer (n)	%	Correct Answer (n)	%	Total Correct Answers (n)	%
**True positive**	**as “0”**	7/10	70%	6/10	60%	0/10	0%	13/30	43.3%
	**as “missing value”**	7/10	70%	6/8	75%	0/3	0%	13/21	61.9%
**False negative**	**as “0”**	3/10	30%	4/10	40%	10/10	100%	17/30	56.6%
	**as “missing value”**	3/10	30%	2/8	25%	3/3	100%	8/21	38.09%
**True negative**	**as “0”**	13 /20	65%	15/20	75%	7/20	35%	35/60	58.3%
	**as “missing value”**	13 /20	65%	15/19	78.9%	7/15	46.6%	35/54	64.8%
**False positive**	**as “0”**	7/20	35%	5/20	25%	13/20	65%	25/60	41.7%
	**as “missing value”**	7/20	35%	4/19	21%	8/15	53.3%	19/54	35.1%
		**% (CI 95%)**		**% (CI 95%)**		**% (CI 95%)**		**% (CI 95%)**	
**Sensitivity**	**as “0”**	70.0% (34.8–93.3%)		60.0% (26.2–87.8%)		0.0% (0–30%)		43.3% (25.5–62.6%)	
	**as “missing value”**	70.00% (34.8–93.3%)		75.00% (34.9–96.8%)		0.00% (0–70.8)		61.90% (38.4–81.9%)	
**Specificity**	**as “0”**	65.0% (40.8–84.6%)		75.0% (50.9–91.3%)		35.0% (15.4–91.3%)		58.3% (44.9–70.9%)	
	**as “missing value”**	65.0% (40.8—84.6%)		78.9% (54.4–93.9%)		46.7% (21.3-		64.8% (50.6–77.3%)	
**Accuracy**	**as “0”**	66.6% (47.2–82.7%)		70.00% (50.6–85.3%		23.3% (9.9–42.3%)		53.33% (42.5–63.9%)	
	**as “missing value”**	66.7% (47.2–82.7%)		77.8% (57.7–91.4%)		38.9% (17.3–64.3)		64.0% (52.1–74.8)	
**PPV**	**as “0”**	50.0% (23–77%)		54.6% (23.4–83.3%)		0.0% (0–14.8%)		34.2% (19.6–51.4)	
	**as “missing value”**	50.0% (23–77%)		60.0% (26.2–87.8)		0.0% (0–36.9%)		40.6% (23.7–59.4%)	
**NPV**	**as “0”**	81.3% (54.4–96%)		78.9% (54.4–93.9%)		41.2% (18.4–67.1%)		67.3% (52.9–79.7%)	
	**as “missing value”**	81.3% (54.4–96%)		88.2% (63.6–98.5%)		70.0% (34.8–93.3%)		81.4% (66.6–91.6%)	

Legend: PPV, Positive Predictive Value; NPV, Negative Predictive Value; CI, confidence interval.

## Data Availability

The raw data supporting the conclusions of this article will be made available by the authors on request.

## Supplementary Materials

The following supporting information can be downloaded at https://www.mdpi.com/article/10.3390/jcm15072638/s1, Tables S1: Diagnosis and intraoral locations of the images included for testing; Table S2A: Responses for question 1 “What is the most probable diagnosis of the observed lesion?” for “Lumps” group; Table S2B: Responses for question 2 “What is the differential diagnosis?” and analysis if the true diagnoses of the question 1 for “Lumps” group; Table S2C: Responses for question 3 “Do you think the lesion is suspicious for oral cancer?”) for “Lumps” group; Table S3A: Responses for question 1 “What is the most probable diagnosis of the observed lesion?” for “OPMD” group; Table S3B: Responses for question 2 “What is the differential diagnosis?” and analysis if the true diagnoses of the question 1 is inside of the given options of differential diagnosis for “OPMD”; Table S3C: Responses for question 3 “Do you think the lesion is suspicious for oral cancer?” for “OPMD” group; Table S4A: Responses for question 1 “What is the most probable diagnosis of the observed lesion?” for “OC” group; Table S4B: Responses for question 2 “What is the differential diagnosis?” and analysis if the true diagnoses of the question 1 is inside of the given options of differential diagnosis for “OC” group; Table S4C: Responses for question 3 “Do you think the lesion is suspicious for oral cancer?” for “OC” group; Table S5: Diagnostic Accuracy metrics analysis obtained from TP, FN, TN, FP results.

## Abbreviations

The following abbreviations are used in this manuscript:

AI	Artificial intelligence
HI	Human intelligence
OPMDs	Oral potentially malignant disorders
LLM	Large language model
OSCC	Oral squamous cell carcinoma
SD	Standard deviation
