# Supplementary material for: Accuracy of AI Tools in the Diagnosis of Benign, Potentially Malignant and Malignant Oral Lesions: A Pilot Study
Source: J Clin Med. 2026 Mar 30;15(7):2638. doi: 10.3390/jcm15072638 (PMC13072891; doi:10.3390/jcm15072638)
Supplement: Supplementary file 1 [file jcm-15-02638-s001.zip › Supplemental table S1.pdf]

Supplemental Table S1 - Diagnosis and intraoral locations of the images included for testing.

Group	Images	Diagnosis	Location
Lumps	Image 1	Squamous cell papilloma	Hard palate
	Image 2	Frictional fibroma (fibrous-epithelial hyperplasia)	Right buccal mucosa
	Image 3	Frictional fibroma (fibrous-epithelial hyperplasia)	Left buccal mucosa
	Image 4	Fibroma (fibrous-epithelial hyperplasia) or fibrous epulis	Upper gingiva
	Image 5	Denture induced fibrous hyperplasia (epulis fissuratum)	Upper left vestibular mucosa
	Image 6	Vascular malformation	Left labial mucosa
	Image 7	Fibroma (fibrous-epithelial hyperplasia) or fibrous epulis	Lower right gingivae
	Image 8	Pyogenic granuloma	Hard palate
	Image 9	Frictional fibroma (fibrous-epithelial hyperplasia)	Tongue
	Image 10	Pyogenic granuloma	Lower left gingivae
OPMDs	Image 11	Leukoplakia	Alveolar mucosa
	Image 12	Leukoplakia with severe dysplasia	Right lateral tongue

	Image 13	Oral Lichen Planus (reticular type)	Right buccal mucosa
	Image 14	Leukoplakia	Ventral tongue
	Image 15	Leukoplakia	Left buccal mucosa
	Image 16	Leukoplakia (with dysplasia)	Right lateral tongue
	Image 17	Leukoplakia	Lower gingivae
	Image 18	Leukoplakia or verrucous leucoplakia	Vestibular mucosa
	Image 19	Oral Lichen Planus	Right buccal mucosa
	Image 20	Leukoplakia	Ventral tongue
Oral Cancers	Image 21	Oral cancer (OSCC)	Alveolar mucosa
	Image 22	Oral cancer (OSCC)	Lower gingival mucosa
	Image 23	Oral cancer (OSCC)	Anterior floor of mouth
	Image 24	Oral cancer (OSCC)	Buccal mucosa right
	Image 25	Oral cancer (OSCC)	Left lateral tongue
	Image 26	Oral cancer (OSCC)	Right floor mouth

	Image 27	Oral cancer (OSCC)	Alveolar mucosa
	Image 28	Oral cancer (mucoepidermoid carcinoma)	Hard palate
	Image 29	Microinvasive carcinoma (OSCC) in a leukoplakia	Ventral tongue
	Image 30	Oral cancer (OSCC)	Ventral tongue

Legend; OSCC, oral squamous cell carcinoma
