# Supplementary material for: Accuracy of AI Tools in the Diagnosis of Benign, Potentially Malignant and Malignant Oral Lesions: A Pilot Study
Source: J Clin Med. 2026 Mar 30;15(7):2638. doi: 10.3390/jcm15072638 (PMC13072891; doi:10.3390/jcm15072638)
Supplement: Supplementary file 1 [file jcm-15-02638-s001.zip › Supplemental Table S2A.pdf]

Supplemental Table S2A

Responses for question 1 "What is the most probable diagnosis of the observed lesion?" for "Lumps" group

Images	Correct Diagnoses	Chatgpt	Correct Answer (0 No/1 Yes)	Gemini	Correct Answer (No/Yes)	Copilot	Correct Answer (No/Yes)	Total Correct Answers
Image 1	Squamous cell papiloma	Oral condyloma acumination (HPV related wart)	0	Wart	1	Oral Squamous Papilloma	1	2
Image 2	Frictional fibroma (fibrous-epithelial hyperplasia)	Mucocele	0	Fibroma	1	Mucocele	0	1
Image 3	Frictional fibroma (fibrous-epithelial hyperplasia)	Traumatic Fibroma	1	Traumatic Fibroma	1	Mucocele	0	2
Image 4	Fibroma (fibrous-epithelial hyperplasia) or fibrous epulis	Pyogenic Granuloma	0	Pyogenic Granuloma	0	Pyogenic Granuloma	0	0
Image 5	Denture induced fibrous hyperplasia (epulis fissuratum)	SCC	0	Unprocessed photographs	Unprocessed photographs	Unprocessed photographs	Unprocessed photographs	0
Image 6	Vascular malformation	Oral Varix	0	Oral Varix	0	Oral Varix	0	0
Image 7	Fibroma (fibrous-epithelial hyperplasia) or Fibrous epulis	Pyogenic Granuloma	0	Pyogenic Granuloma	0	Unprocessed photographs	Unprocessed photographs	0
Image 8	Pyogenic granuloma	Pyogenic Granuloma	1	Pyogenic Granuloma	1	Unprocessed photographs	Unprocessed photographs	2
Image 9	Frictional fibroma (fibrous-epithelial hyperplasia)	Fibroma	1	Benign migratory glossitis	0	Fibroma	1	2

Image 10	Pyogenic granuloma	Pericoronitis (acute pericoronitis)	0	Osteonecrosis of the Jaw (ONJ)	0	Gengivitis	0	0
TOTAL	**(0 - 10)**		**3**		**4**		**2**	**9**
TOTAL (%)	**(Considering "unprocessed photographs" as "0")**		**30%**		**40%**		**20%**	**30%**
TOTAL (%)	**(Considering "unprocessed photographs" as "missing value")**		**30%**		**44%**		**28%**	**34.6%**
