# Supplementary material for: Accuracy of AI Tools in the Diagnosis of Benign, Potentially Malignant and Malignant Oral Lesions: A Pilot Study
Source: J Clin Med. 2026 Mar 30;15(7):2638. doi: 10.3390/jcm15072638 (PMC13072891; doi:10.3390/jcm15072638)
Supplement: Supplementary file 1 [file jcm-15-02638-s001.zip › Supplemental Table S2B.pdf]

Supplemental Table S2B: Responses for question 2 "What is the differential diagnosis?" and analysis if the true diagnoses of the question 1 for "Lumps" group

Images	Correct Diagnosis	ChatGPT			Gemini			CoPilot		
		**Top 5 of Differential diagnoses**	**Include the right diagnosis in the differential diagnoses?**	**Total of right diagnosis and included in Differential Diagnosis**	**Top 5 of Differential diagnosis**	**Include the right diagnosis in the differential diagnoses?**	**Total of right diagnosis and included in Differential Diagnosis**	**Top 5 of Differential diagnosis**	**Include the right diagnosis in the differential diagnoses?**	**Total of right diagnosis and included in Differential Diagnosis**
Image 1	Squamous cell papilloma	1. Squamous papilloma<br>2. Verruca vulgaris (common wart)<br>3. Focal epithelial hyperplasia<br>4. Verrucous carcinoma<br>5. Multifocal epithelial hyperplasia due to immune suppression (e.g., HIV-related)	1	1	1. Verruciform Xanthoma<br>2. Benign Fibrous Hyperplasia<br>3. Squamous Cell Carcinoma<br>4. Condyloma Acuminatum<br>5. Papilloma (Generalized term)<br>6. others	Not applicable	1	1. Verruca Vulgaris (Common Wart)<br>2. Condyloma Acuminatum<br>3. Focal Epithelial Hyperplasia<br>4. Fibroma<br>5. Pyogenic Granuloma<br>6. Squamous Cell Carcinoma	Not applicable	1
Image 2	Frictional fibroma (fibrous-epithelial hyperplasia)	1. Fibroma<br>2. Salivary Gland Neoplasm (e.g., Pleomorphic Adenoma)<br>3. Lipoma<br>4. Traumatic Granuloma<br>5. Hemangioma<br>6. Epidermoid or Dermoid Cyst	1	1	1. Mucocele<br>2. Lipoma<br>3. Benign Minor Salivary Gland Tumor (e.g., Pleomorphic Adenoma)<br>4. Neurofibroma/Schwannoma<br>5. Squamous Cell Carcinoma	Not applicable	1	1. Fibroma<br>2. Pyogenic Granuloma<br>3. Lipoma<br>4. Salivary Gland Tumor (e.g., pleomorphic adenoma)<br>5. Hemangioma<br>6. Lymphangioma	1	1
Image 3	Frictional fibroma (fibrous-epithelial hyperplasia)	1. Squamous Papilloma<br>2. Mucocele<br>3. Pyogenic Granuloma<br>4. Peripheral Ossifying Fibroma<br>5. Minor Salivary Gland Tumor (e.g., Pleomorphic Adenoma)<br>6. Oral Verruca Vulgaris	Not applicable	1	1. Mucocele<br>2. Lipoma<br>3. Benign Minor Salivary Gland Tumor (e.g., Pleomorphic Adenoma)<br>4. Neurofibroma/Schwannoma<br>5. Granular Cell Tumor<br>6. Low-grade SCC	Not applicable	1	1. Fibroma<br>2. Trauma-Induced Lesion<br>3. Aphthous Ulcer<br>4. Infectious Lesion<br>5. Squamous Papilloma<br>6. others	1	1
Image 4	Fibroma (fibrous-epithelial	1. Peripheral Giant Cell Granuloma<br>2. Peripheral Ossifying Fibroma<br>3. Parulis (Gum boil)	1	1	1. Peripheral Giant Cell Granuloma<br>2. Peripheral Ossifying Fibroma	1	1	1. Peripheral Giant Cell granuloma<br>2. Fibroma<br>3. Peripheral Ossifying Fibroma	1	1

	hyperplasia) or Fibrous epulis	4. Fibroma<br>5. Hemangioma<br>6. Kaposi's Sarcoma			3. Inflammatory Fibrous Hyperplasia (Irritation Fibroma)<br>4. Parulis (Gum Boil/Abscess)<br>5. Squamous Cell Carcinoma			4. Kaposi's Sarcoma<br>5. Hemangioma<br>6. others		
Image 5	Denture induced fibrous hyperplasia (epulis fissuratum)	1. Oral Squamous Cell Carcinoma<br>2. Traumatic Ulcer<br>3. Necrotizing Sialometaplasia<br>4. Deep Fungal Infection (e.g., Histoplasmosis, Mucormycosis)<br>5. Major Aphthous Ulcer<br>6. others	0	0	unprocessed photographs	unprocessed photographs	unprocessed photographs	unprocessed photographs	unprocessed photographs	unprocessed photographs
Image 6	Vascular malformation	1. Hemangioma<br>2. Mucocele<br>3. Hematoma<br>4. Angioleiomyoma<br>5. Kaposi Sarcoma<br>6. Melanotic Macule or Melanoma	1	1	1. Hemangioma<br>2. Pyogenic Granuloma<br>3. Melanoma<br>4. Peripheral Giant Cell Granuloma<br>5. Mucocele/Ranula (Thrombosed)	1	1	1. Hemangioma<br>2. Angiokeratoma<br>3. Oral Melanotic Macule<br>4. Amalgam Tattoo<br>5. Kaposi Sarcoma<br>6. Oral Melanoma	1	1
Image 7	Fibroma (fibrous-epithelial hyperplasia) or Fibrous epulis	1. Peripheral Giant Cell Granuloma<br>2. Peripheral Ossifying Fibroma<br>3. Irritation Fibroma<br>4. Hemangioma<br>5. Kaposi's Sarcoma	1	1	1. Peripheral Ossifying Fibroma<br>2. Peripheral Giant Cell Granuloma<br>3. Irritation Fibroma<br>4. Parulis / Draining Sinus Tract<br>5. Squamous Cell Carcinoma	1	1	unprocessed photographs	unprocessed photographs	unprocessed photographs
Image 8	Pyogenic granuloma	1. Peripheral Giant Cell Granuloma<br>2. Peripheral Ossifying Fibroma<br>3. Fibroma<br>4. Parulis (Gum Boil)<br>5. Peripheral Ameloblastoma<br>6. Kaposi's Sarcoma	Not applicable	1	1. Peripheral Ossifying Fibroma<br>2. Peripheral Giant Cell Granuloma<br>3. Irritation Fibroma<br>4. Gingival Abscess / Parulis<br>5. Squamous Cell Carcinoma<br>6. Hemangioma	Not applicable	1	unprocessed photographs	unprocessed photographs	unprocessed photographs
Image 9	Frictional fibroma (fibrous-epithelial hyperplasia)	1. Mucocele<br>2. Papilloma (Squamous Papilloma)<br>3. Granular Cell Tumor<br>4. Hemangioma / V. malformation<br>5. Neurofibroma or Schwannoma<br>6. Pyogenic Granuloma	Not applicable	1	Traumatic Fibroma<br>2. Granular Cell Tumor<br>3. Pyogenic Granuloma<br>4. Oral Papilloma<br>5. Early Squamous Cell Carcinoma	1	1	1. Pyogenic Granuloma<br>2. Peripheral Giant Cell Granuloma<br>3. Lipoma<br>4. Squamous Papilloma<br>5. Neurofibroma<br>6. Fibrosarcoma	Not applicable	1

Image 10	Pyogenic granuloma	1. Periodontal Abscess<br>2. Traumatic Ulcer or Fibroma<br>3. Eruption Cyst<br>4. Necrotizing Ulcerative Gingivitis<br>5. Oral Squamous Cell Carcinoma<br>6. Aphthous Ulcer	0	0	1. Localized Osteomyelitis<br>2. Sequestrum<br>3. Healing Complication Post-Extraction/Trauma<br>4. Necrotic Lesion<br>5. Malignancy	0	0	1. Periodontal disease<br>2. Dental abscess<br>3. Pericoronitis<br>4. Traumatic gingival lesion<br>5. Leukoplakia<br>6. Necrotizing periodontal disease	0	0
TOTAL	**(0 - 10)**		**5**	**8**		**4**	8		**4**	6
TOTAL (%)	**(Considering** " unprocessed photographs " **as "0")**		**71%**	**80%**		**(4/6) 66.6%**	**(8/10) 80%**		**(4/8) 50%**	**(6/10) 60%**
TOTAL (%)	**(Considering** " unprocessed photographs " **as "missing value")**		**71%**	**80%**		**(4/5) 80%**	**(8/9) 88.8%**		(4/5) **80%**	**(6/7) 85.7%**
