# Supplementary material for: Accuracy of AI Tools in the Diagnosis of Benign, Potentially Malignant and Malignant Oral Lesions: A Pilot Study
Source: J Clin Med. 2026 Mar 30;15(7):2638. doi: 10.3390/jcm15072638 (PMC13072891; doi:10.3390/jcm15072638)
Supplement: Supplementary file 1 [file jcm-15-02638-s001.zip › Supplemental Table S2C.pdf]

Supplemental Table S2C - Responses for question 3 "Do you think the lesion is suspicious for oral cancer?”) for "Lumps" group

Images	Correct Diagnosis	Chatgpt	Correct Answer (0 No/1 Yes)	Gemini	Correct Answer (No/Yes)	Copilot	Correct Answer (No/Yes)	Total Correct Answers
Image 1	no	no	1	no	1	no	1	3
Image 2	no	no	1	no	1	no	1	3
Image 3	no	no	1	no	1	No answer	0	2
Image 4	no	no	1	no	1	no	1	3
Image 5	no	yes	0	unprocessed photographs	unprocessed photographs	unprocessed photographs	unprocessed photographs	0
Image 6	no	no	1	no	1	no	1	3
Image 7	no	no	1	no	1	unprocessed photographs	unprocessed photographs	2
Image 8	no	no	1	no	1	unprocessed photographs	unprocessed photographs	2
Image 9	no	no	1	no	1	no	1	3
Image 10	no	no	1	no	1	no	1	3
TOTAL	**(0 - 10)**		**9**		**9**		**6**	**24**
TOTAL (%)	**(Considering** " unprocessed photographs " **as "0")**		**90%**		**90%**		**60%**	**80%**
TOTAL (%)	**(Considering** " unprocessed photographs " **as "missing value")**		**90%**		**100%**		**85%**	**92.3%**
