# Supplementary material for: Accuracy of AI Tools in the Diagnosis of Benign, Potentially Malignant and Malignant Oral Lesions: A Pilot Study
Source: J Clin Med. 2026 Mar 30;15(7):2638. doi: 10.3390/jcm15072638 (PMC13072891; doi:10.3390/jcm15072638)
Supplement: Supplementary file 1 [file jcm-15-02638-s001.zip › Supplemental Table S3A.pdf]

Supplemental Table S3A - Responses for question 1 "What is the most probable diagnosis of the observed lesion?" for "OPMD" group

Images	Correct Diagnosis	ChatGPT	Correct Answer (0 No/1 Yes)	Gemini	Correct Answer (No/Yes)	Copilot	Correct Answer (No/Yes)	Total Correct Answers
Image 11	Leukoplakia	Oral leukoplakia	1	Alveolar osteitis (dry socket)	0	Oral leukoplakia	1	2
Image 12	Leukoplakia with severe dysplasia	Oral candidiasis (chronic hyperplastic type)	0	Oral candidiasis (pseudomembranous candidiasis/thrush)	0	Oral candidiasis	0	0
Image 13	OLP (reticular type)	Lichen planus (reticular type)	1	Lichen planus	1	Oral leukoplakia	0	2
Image 14	Leukoplakia	Oral leukoplakia	1	Oral candidiasis	0	unprocessed photographs	unprocessed photographs	1
Image 15	Leukoplakia	Oral leukoplakia	1	Morsicatio buccarum (chronic cheek biting)	0	Oral leukoplakia	1	2
Image 16	Leukoplakia (with dysplasia)	Oral leukoplakia	1	Oral leukoplakia	1	unprocessed photographs	unprocessed photographs	2
Image 17	Leukoplakia	Oral leukoplakia	1	Chemical/irritant induced oral mucosal peeling	0	Oral leukoplakia	1	2
Image 18	Leukoplakia or verrucous leucoplakia/PVL	Oral hairy leukoplakia	0	Non-homogeneous leukoplakia	0	Oral leukoplakia	1	1
Image 19	OLP	Lichen planus (reticular form)	1	Lichen planus	1	Lichen planus	1	3

Image 20	Leukoplakia	Oral leukoplakia	1	Oral leukoplakia	1	Oral candidiasis	0	2
TOTAL	**(0 - 10)**		**8**		**4**		**5**	**17**
TOTAL (%)	**(Considering** " unprocessed photographs " **as "0")**		**80%**		**40%**		**50%**	**56.6%**
TOTAL (%)	**(Considering** " unprocessed photographs " **as "missing value")**		**80%**		**40%**		**62%**	**60.7%**
