# Supplementary material for: Accuracy of AI Tools in the Diagnosis of Benign, Potentially Malignant and Malignant Oral Lesions: A Pilot Study
Source: J Clin Med. 2026 Mar 30;15(7):2638. doi: 10.3390/jcm15072638 (PMC13072891; doi:10.3390/jcm15072638)
Supplement: Supplementary file 1 [file jcm-15-02638-s001.zip › Supplemental Table S3B.pdf]

Supplemental Table S3B - Responses for question 2 "What is the differential diagnosis?" and analysis if the true diagnoses of the question 1 is inside of the given options of differential diagnosis for “OPMD" group

Image	Correct Diagnosis	ChatGPT			Gemini			CoPilot		
		**Top 5 of Differential diagnoses**	**Include the right diagnosis in the differential diagnoses?**	**Total of right diagnosis and included in Differential Diagnosis**	**Top 5 of Differential diagnosis**	**Include the right diagnosis in the differential diagnoses?**	**Total of right diagnosis and included in Differential Diagnosis**	**Top 5 of Differential diagnosis**	**Include the right diagnosis in the differential diagnoses?**	**Total of right diagnosis and included in Differential Diagnosis**
11	Leukoplakia	1. Chronic Hyperplastic Candidiasis<br>2. Frictional Keratosis<br>3. Oral Lichen Planus (Plaque-like type)<br>4. Oral Squamous Cell Carcinoma 5. Nicotinic Stomatitis (Smoker's Palate)	Not applicable	1	1. Post-operative Infection<br>2. Foreign Body/Retained Root Fragment<br>3. Trauma to Adjacent Structures<br>4. Osteomyelitis<br>5. Referred Pain<br>6. Temporomandibular Joint (TMJ)	0	0	1. Oral lichen planus<br>2. Candidiasis (oral thrush)<br>3,. Frictional keratosis<br>4. Hairy leukoplakia<br>5. Squamous cell carcinoma<br>6. Lupus erythematosus	Not applicable	1
12	Leukoplakia with severe dysplasia	1. Chronic Hyperplastic Candidiasis<br>2. Oral Hairy Leukoplakia<br>3. Oral Lichen Planus (Plaque-Type)	1	1	1. Leukoplakia<br>2. Lichen Planus (Reticular or Plaque-like)<br>3. Hairy Leukoplakia<br>4. Geographic Tongue	1	1	1. Leukoplakia<br>2. Lichen planus<br>3. Oral hairy leukoplakia<br>4. Burn or irritation lesion	1	1

		4. Leukoplakia (Idiopathic/Precancerous)<br>5. Squamous Cell Carcinoma<br>6. others			5. Chemical/Thermal Burn (Acute)<br>6. Factitial Lesion			5. Squamous cell carcinoma<br>6. Syphilitic plaque		
13	OLP (reticular type)	1. Oral Lichenoid Reaction (OLR)<br>2. Lupus Erythematosus<br>3. Chronic Hyperplastic Candidiasis<br>4. Leukoplakia<br>5. Graft-versus-Host Disease<br>6. Frictional Keratosis	Not applicable	1	1. Lichenoid Reaction<br>2. Chronic Hyperplastic Candidiasis<br>3. Leukoplakia<br>4. Hairy Leukoplakia<br>5. Frictional Keratosis<br>6. Squamous Cell Carcinoma (Verrucous)	Not applicable	1	1. Oral lichen planus<br>2. Hyperkeratosis (mechanical trauma)<br>3. Candidiasis (chronic hyperplastic type)<br>4. Squamous cell carcinoma<br>5. Leukoedema<br>6. White sponge nevus	1	1
I 4	Leukoplakia	1. Oral Lichen Planus (OLP)<br>2. Chronic Hyperplastic Candidiasis<br>3. Frictional Keratosis<br>4. Oral Squamous Cell Carcinoma (SCC)<br>5. Discoid Lupus Erythematosus (DLE)<br>6. White Sponge Nevus	Not applicable	1	1. Leukoplakia<br>2. Plaque-like Oral Lichen Planus<br>3. Hairy Leukoplakia<br>4. Chronic Traumatic/Frictional Keratosis<br>5. Geographic Tongue (Benign Migratory Glossitis - Atypical presentation)	1	1	unprocessed photographs	unprocessed photographs	unprocessed photographs

15	Leukoplakia	1. Frictional Keratosis<br>2. Oral Lichen Planus<br>3. Chronic Hyperplastic Candidiasis<br>4. Oral Hairy Leukoplakia<br>5. Verrucous Carcinoma or Early Squamous Cell Carcinoma	Not applicable	1	1. Leukoplakia<br>2. Chronic Hyperplastic Candidiasis<br>3. Oral Lichen Planus<br>4. Squamous Cell Carcinoma (White/Verrucous Forms)	1	1	1. Oral lichen planus<br>2. Chronic hyperkeratosis<br>3. Candidiasis (Hyperplastic type)<br>4. Squamous cell carcinoma<br>5. Frictional keratosis<br>6. others	Not applicable	1
16	Leukoplakia (with dysplasia)	1. Oral Lichen Planus (Reticular Form)<br>2. Chronic Hyperplastic Candidiasis<br>3. Frictional Keratosis<br>4. Oral Hairy Leukoplakia<br>5. Early Squamous Cell Carcinoma (SCC)	Not applicable	1	1. Lichen Planus<br>2. Candidiasis (Chronic Hyperplastic)<br>3. Squamous Cell Carcinoma<br>4. Nicotinic Stomatitis<br>5. Frictional Keratosis	Not applicable	1	unprocessed photographs	unprocessed photographs	unprocessed photographs
17	Leukoplakia	1. Frictional Keratosis<br>2. Oral Lichen Planus (Reticular Type)<br>3. Oral Hairy Leukoplakia<br>4. Chronic Hyperplastic Candidiasis<br>5. Discoid Lupus Erythematosus	Not applicable	1	1. Morsicatio Buccarum<br>2. Leukoedema<br>3. Oral Lichen Planus<br>4. Candidiasis<br>5. Thermal Burn<br>6. Desquamative Gingivitis	0	0	1. Frictional keratosis<br>2. Oral lichen planus (reticular type)<br>3. Chronic hyperplastic candidiasis<br>4. White sponge nevus	Not applicable	1

								5. Squamous cell carcinoma (early-stage)		
18	Leukoplakia or verrucous leucoplakia/PVL	1. Frictional Keratosis<br>2. Idiopathic Leukoplakia<br>3. Lichen Planus (Plaque-type)<br>4. Oral Candidiasis (Chronic Hyperplastic)<br>5. Proliferative Verrucous Leukoplakia	1	1	1. Squamous Cell Carcinoma (Exophytic/Verrucous Type)<br>2. Chronic Hyperplastic Candidiasis<br>3. Oral Lichen Planus (Plaque-like variant)<br>4. Frictional Keratosis / Benign Alveolar Ridge Keratosis (BARK)	0	0	1. Frictional Keratosis<br>2. Oral Lichen Planus<br>3. Chronic Hyperplastic Candidiasis<br>4. Squamous Cell Carcinoma<br>5. White Sponge Nevus<br>6. others	Not applicable	1
19	OLP	1. Frictional Keratosis (Chronic Irritation)<br>2. Leukoplakia<br>3. Chronic Hyperplastic Candidiasis<br>4. Discoid Lupus Erythematosus (DLE)<br>5. Oral Hairy Leukoplakia<br>6. Smokeless Tobacco Keratosis	Not applicable	1	1. Erythroleukoplakia<br>2. Lichenoid Reaction<br>3. Chronic Hyperplastic Candidiasis<br>4. Oral Squamous Cell Carcinoma (OSCC)<br>5. Lupus Erythematosus<br>6. Chronic Ulcerative Stomatitis (CUS)	Not applicable	1	1. Leukoplakia<br>2. Oral Candidiasis (Thrush)<br>3. Mucosal Dysplasia<br>4. Erythroplakia<br>5. Traumatic Keratosis<br>6. others	Not applicable	1
20	Leukoplakia	Proliferative Verrucous Leukoplakia<br>2. Oral Lichen Planus (Plaque-Type)<br>3. Chronic Hyperplastic Candidiasis	Not applicable	1	1. Proliferative Verrucous Leukoplakia<br>2. Verrucous Carcinoma<br>3. Oral Squamous Cell Carcinoma (OSCC)	Not applicable	1	1. Leukoplakia<br>2. Lichen Planus<br>3. Erythroplakia<br>4. Traumatic Lesion	1	1

		4. Frictional Keratosis (Benign Reactive)<br>5. Early Squamous Cell Carcinoma<br>6. Smokeless Tobacco Keratosis			4. Oral Lichen Planus (Plaque-like form)<br>5. Frictional Keratosis/Hyperkeratosis			5. Nicotine Stomatitis		
TOTAL	**(0 a 10)**		**2**	**10**		**3**	**7**		**3**	**8**
TOTAL (%)	**(Considering** " unprocessed photographs " **as "0")**		**(2/2)**<br>**100%**	**(10/10)**<br>**100%**		**(3/6)**<br>**50%**	**(7/10)**<br>**70%**		**3/5**<br>**60%**	**8/10**<br>**80%**
TOTAL (%)	**(Considering** " unprocessed photographs " **as "missing value")**		**(2/2)**<br>**100%**	**(10/10)**<br>**100%**		**(3/6)**<br>**50%**	**(7/10)**<br>**70%**		**3/3**<br>**100%**	**8/8**<br>**100%**
