# Supplementary material for: Accuracy of AI Tools in the Diagnosis of Benign, Potentially Malignant and Malignant Oral Lesions: A Pilot Study
Source: J Clin Med. 2026 Mar 30;15(7):2638. doi: 10.3390/jcm15072638 (PMC13072891; doi:10.3390/jcm15072638)
Supplement: Supplementary file 1 [file jcm-15-02638-s001.zip › Supplemental Table S3C.pdf]

Supplemental Table S3C - Responses for question 3 "Do you think the lesion is suspicious for oral cancer?" for "OPMD" group

Images	Correct Diagnosis	Chatgpt	Correct Answer (0 No/1 Yes)	Gemini	Correct Answer (No/Yes)	Copilot	Correct Answer (No/Yes)	Total Correct Answers
Image 11	no	yes	0	no	1	yes	0	1
Image 12	yes	yes	1	no	0	no	0	1
Image 13	no	no	1	no	1	yes	0	2
Image 14	no	yes	0	no	1	unprocessed photographs	unprocessed photographs	1
Image 15	no	yes	0	no	1	yes	0	1
Image 16	yes	yes	1	yes	1	unprocessed photographs	unprocessed photographs	2
Image 17	no	yes	0	no	1	yes	0	1
Image 18	no	yes	0	yes	0	yes	0	0
Image 19	no	no	1	yes	0	No answer	0	1
Image 20	no	yes	0	yes	0	no	1	1
TOTAL	**(0 - 10)**		**4**		**6**		**1**	**11**
TOTAL (%)	**(Considering** " unprocessed photographs " **as "0")**		**40%**		**60%**		**10%**	**36.6%**
TOTAL (%)	**(Considering** " unprocessed photographs " **as "missing value")**		**40%**		**60%**		**12.5%**	**39.2%**
