# Supplementary material for: Accuracy of AI Tools in the Diagnosis of Benign, Potentially Malignant and Malignant Oral Lesions: A Pilot Study
Source: J Clin Med. 2026 Mar 30;15(7):2638. doi: 10.3390/jcm15072638 (PMC13072891; doi:10.3390/jcm15072638)
Supplement: Supplementary file 1 [file jcm-15-02638-s001.zip › Supplemental Table S4A.pdf]

Supplemental Table S4A - Responses for question 1 "What is the most probable diagnosis of the observed lesion?" for "OC" group

Images	Correct Diagnosis	Chatgpt	Correct Answer (0 No/1 Yes)	Gemini	Correct Answer (No/Yes)	Copilot	Correct Answer (No/Yes)	Total Correct AnswerS
Image 21	Oral cancer (OSCC)	SCC	1	SCC	1	unprocessed photographs	unprocessed photographs	2
Image 22	Oral cancer (OSCC)	Oral leukoplakia	0	Varrucous carcinoma	0	Oral candidiasis	0	0
Image 23	Oral cancer (OSCC)	Oral leukoplakia	0	Non-homogeneous leukoplakia	0	Oral candidiasis	0	0
Image 24	Oral cancer (OSCC)	SCC	1	Not evaluated	unprocessed photographs	unprocessed photographs	unprocessed photographs	1
Image 25	Oral cancer (OSCC)	Osteonecrosis of the Jaw (ONJ)	0	Osteonecrosis of the Jaw (ONJ)	0	unprocessed photographs	unprocessed photographs	0
Image 26	Oral cancer (OSCC)	SCC	1	Wart	0	unprocessed photographs	unprocessed photographs	1
Image 27	Oral cancer (OSCC)	SCC	1	unprocessed photographs	unprocessed photographs	unprocessed photographs	unprocessed photographs	1
Image 28	Oral cancer (mucoepidermoid carcinoma)	Palatal mucocele or palatal salivary gland cyst	0	Torus palatinus	0	Torus palatinus	0	0
Image 29	Microinvasive carcinoma (OSCC) in a leukoplakia	Oral leukoplakia	0	Lichen planus	0	unprocessed photographs	unprocessed photographs	1
Image 30	Oral cancer (OSCC)	Ranula	0	Canker sore (aphthous ulcer)	0	unprocessed photographs	unprocessed photographs	0
TOTAL	**(0 - 10)**		**4**		**1**		**0**	**6**

TOTAL (%)	**(Considering** " unprocessed photographs " **as "0")**	**40%**		**10%**		**0%**	**20%**
TOTAL (%)	**(Considering** " unprocessed photographs " **as "missing value")**	**40%**		**12.5%**		**0%**	**28.5%**
