# Supplementary material for: Accuracy of AI Tools in the Diagnosis of Benign, Potentially Malignant and Malignant Oral Lesions: A Pilot Study
Source: J Clin Med. 2026 Mar 30;15(7):2638. doi: 10.3390/jcm15072638 (PMC13072891; doi:10.3390/jcm15072638)
Supplement: Supplementary file 1 [file jcm-15-02638-s001.zip › Supplemental Table S4B.pdf]

**Supplemental Table S4B -** Responses for question 2 "What is the differential diagnosis?" and analysis if the true diagnoses of the question 1 is inside of the given options of differential diagnosis for "OC" group

| Images | Correct Diagnosis | ChatGPT | | | Gemini | | | CoPilot | | |
|---|---|---|---|---|---|---|---|---|---|---|
| | | **Top 5 of Differential diagnoses** | **Include the right diagnosis in the differential diagnoses?** | **Total of right diagnosis and included in Differential Diagnosis** | **Top 5 of Differential diagnosis** | **Include the right diagnosis in the differential diagnoses?** | **Total of right diagnosis and included in Differential Diagnosis** | **Top 5 of Differential diagnosis** | **Include the right diagnosis in the differential diagnoses?** | **Total of right diagnosis and included in Differential Diagnosis** |
| 21 | Oral cancer (OSCC) | 1. Chronic Traumatic Ulcer<br>2. Deep Fungal Infection<br>3. Tuberculosis (Oral TB)<br>4. Syphilitic Chancre or Mucous Patch<br>5. Lymphoma (e.g., Non-Hodgkin)<br>6. others | Not applicable | 1 | 1. Chronic Traumatic Ulcer<br>2. Deep Fungal Infection<br>3. Granulomatous Inflammation<br>4. Major Aphthous Ulcer<br>5. Syphilitic Chancre<br>6. Tuberculosis Ulcer | Not applicable | 1 | unprocessed photographs | unprocessed photographs | unprocessed photographs |
| 22 | Oral cancer (OSCC) | 1. Chronic Hyperplastic Candidiasis<br>2. Oral Lichen Planus (Plaque-type)<br>3. Frictional (Traumatic) Keratosis | 1 | 1 | 1. Proliferative Verrucous Leukoplakia<br>2. Squamous Cell Carcinoma (Exophytic/Verrucous Type) | 1 | 1 | 1. Leukoplakia<br>2. Lichen Planus<br>3. Aphthous Ulcers<br>4. Erythroplakia | 1 | 1 |

| | | | | | | | | | | |
|---|---|---|---|---|---|---|---|---|---|---|
| | | 4. Smokeless Tobacco Keratosis<br>5. Early Squamous Cell Carcinoma (SCC)<br>6. Lupus Erythematosus | | | 3. Chronic Hyperplastic Candidiasis<br>4. Oral Papilloma<br>5. Frictional Keratosis / Hyperkeratosis | | | 5. Pemphigus Vulgaris<br>6. others | | |
| 23 | Oral cancer (OSCC) | 1. Oral Squamous Cell Carcinoma (SCC)<br>2. Chronic Hyperplastic Candidiasis<br>3. Proliferative Verrucous Leukoplakia<br>4. Oral Lichen Planus<br>5. Frictional Keratosis / Chronic Trauma<br>6. Verrucous Carcinoma | 1 | 1 | 1. Squamous Cell Carcinoma (Exophytic/Verrucous Type)<br>2. Verrucous Carcinoma<br>3. Chronic Hyperplastic Candidiasis<br>4. Lichen Planus (Plaque-like variant)<br>5. Frictional Keratosis | 1 | 1 | 1. Oral Lichen Planus<br>2. Leukoplakia<br>3. Erythematous Candidiasis<br>4. Contact Stomatitis<br>5. Squamous Cell Carcinoma<br>6. others | 1 | 1 |
| 24 | Oral cancer (OSCC) | 1. Proliferative Verrucous Leukoplakia<br>2. Chronic Traumatic Ulcer<br>3. Oral Candidiasis<br>4. Oral Lichen Planus (Erosive Type)<br>5. Verrucous Carcinoma<br>6. others | Not applicable | 1 | unprocessed photographs | unprocessed photographs | unprocessed photographs | unprocessed photographs | unprocessed photographs | unprocessed photographs |

| | | | | | | | | | | |
|---|---|---|---|---|---|---|---|---|---|---|
| 25 | Oral cancer (OSCC) | 1. Osteoradionecrosis (ORN)<br>2. Chronic Osteomyelitis<br>3. Oral Squamous Cell Carcinoma (OSCC)<br>4. Necrotizing Ulcerative Gingivitis<br>5. Traumatic Ulcer with Infection | 1 | 1 | 1. Squamous Cell Carcinoma (SCC)<br>2. Osteomyelitis<br>3. Traumatic Ulcer with Bone Exposure<br>4. Oral Ulceration with Bone Sequestration<br>5. Glandular Odontogenic Cyst (GOC) / Periapical or Periodontal Abscess | 1 | 1 | unprocessed photographs | unprocessed photographs | unprocessed photographs |
| 26 | Oral cancer (OSCC) | 1. Chronic Traumatic Ulcer<br>2. Infectious Ulcer<br>3. Major Aphthous Ulcer<br>4. Autoimmune Conditions<br>5. Eosinophilic Ulcer<br>6. others | Not applicable | 1 | 1. Pyogenic Granuloma<br>2. Traumatic Ulcer<br>3. Squamous Cell Carcinoma<br>4. Major Aphthous Ulcer<br>5. Inflammatory Fibrous Hyperplasia<br>6. Necrotizing Sialometaplasia | 1 | 1 | unprocessed photographs | unprocessed photographs | unprocessed photographs |
| 27 | Oral cancer (OSCC) | 1. Erythroplakia<br>2. Chronic Traumatic Ulcer<br>3. Chronic Hyperplastic Candidiasis<br>4. Lichen Planus (Erosive type)<br>5. Oral Lichenoid Reaction (OLR)<br>6. others | Not applicable | 1 | unprocessed photographs | unprocessed photographs | unprocessed photographs | unprocessed photographs | unprocessed photographs | unprocessed photographs |

| | | | | | | | | | | |
|---|---|---|---|---|---|---|---|---|---|---|
| 28 | Oral cancer (mucoepidermoid carcinoma) | 1. Palatal Abscess<br>2. Torus Palatinus<br>3. Pleomorphic Adenoma<br>4. Fibroma<br>5. Median Palatal Cyst<br>6. Mucoepidermoid Carcinoma | 1 | 1 | 1. Inflammatory fibroma<br>2. Minor salivary gland tumor (benign or malignant)<br>3. Abscess<br>4. Mucocele<br>5. Nasopalatine duct cyst | 0 | 0 | 1. Fibroma<br>2. Mucous Retention Cyst<br>3. Squamous Cell Carcinoma<br>4. Pleomorphic Adenoma<br>5. Pyogenic Granuloma<br>6. others | 1 | 1 |
| 29 | Microinvasive carcinoma (OSCC) in a leukoplakia | 1. Frictional Keratosis<br>2. Oral Lichen Planus (OLP)<br>3. Chronic Hyperplastic Candidiasis<br>4. Proliferative Verrucous Leukoplakia<br>5. Early Squamous Cell Carcinoma<br>6. Oral Submucous Fibrosis (OSMF) | Not applicable | 1 | 1. Lichenoid Reaction<br>2. Lupus Erythematosus<br>3. Hyperplastic Candidiasis<br>4. Leukoplakia<br>5. Psoriasis (oral manifestations)<br>6. others | 1 | 1 | unprocessed photographs | unprocessed photographs | unprocessed photographs |
| 30 | Oral cancer (OSCC) | 1. Mucocele (Less likely in this location)<br>2. Dermoid/Epidermoid Cyst<br>3. Salivary Gland Tumor | 0 | 0 | 1. Traumatic Ulcer<br>2. Herpetic Ulceration<br>3. Early squamous Cell Carcinoma<br>4. Necrotizing Sialometaplasia | 1 | 1 | unprocessed photographs | unprocessed photographs | unprocessed photographs |

| | | | | | | | | | | |
|---|---|---|---|---|---|---|---|---|---|---|
| | | 4. Hemangioma or V. Malformation<br>5. Lymphoepithelial Cyst<br>6. Sialolithiasis (Salivary Stone) | | | 5. Mucocele (ruptured/ulcerated) | | | | | |
| TOTAL | **(0 - 10)** | | **4** | **9** | | **6** | **7** | | **3** | **3** |
| TOTAL (%) | **(Considering** " unprocessed photographs " **as "0")** | | **4/5**<br>**80%** | **9/10**<br>**90%** | | **6/9**<br>**66%** | **7/10**<br>**70%** | | **3/10**<br>**30%** | **3/10**<br>**30%** |
| TOTAL (%) | **(Considering** " unprocessed photographs " **as "missing value")** | | **4/5**<br>**80%** | **9/10**<br>**90%** | | **6/7**<br>**85%** | **7/8**<br>**87.5%** | | **3/3**<br>**100%** | **3/3**<br>**100%** |
