# Supplementary material for: Accuracy of AI Tools in the Diagnosis of Benign, Potentially Malignant and Malignant Oral Lesions: A Pilot Study
Source: J Clin Med. 2026 Mar 30;15(7):2638. doi: 10.3390/jcm15072638 (PMC13072891; doi:10.3390/jcm15072638)
Supplement: Supplementary file 1 [file jcm-15-02638-s001.zip › Supplemental Table S4C.pdf]

Supplemental Table S4C - Responses for question 3 "Do you think the lesion is suspicious for oral cancer?" for "OC" group

Images	Correct Diagnosis	Chatgpt	Correct Answer (0 No/1 Yes)	Gemini	Correct Answer (No/Yes)	Copilot	Correct Answer (No/Yes)	Total Correct Answers
Image 21	yes	yes	1	yes	1	unprocessed photographs	unprocessed photographs	2
Image 22	yes	yes	1	yes	1	no	0	2
Image 23	yes	yes	1	yes	1	no	0	2
Image 24	yes	yes	1	unprocessed photographs	unprocessed photographs	unprocessed photographs	unprocessed photographs	1
Image 25	yes	no	0	yes	1	unprocessed photographs	unprocessed photographs	1
Image 26	yes	yes	1	yes	1	unprocessed photographs	unprocessed photographs	2
Image 27	yes	yes	1	unprocessed photographs	unprocessed photographs	unprocessed photographs	unprocessed photographs	1
Image 28	yes	no	0	no	0	no	0	0
Image 29	yes	yes	1	yes	1	unprocessed photographs	unprocessed photographs	2
Image 30	yes	no	0	no	0	unprocessed photographs	unprocessed photographs	0
TOTAL	**(0 - 10)**		**7**		**6**		**0**	**13**

TOTAL (%)	**(Considering** " unprocessed photographs " **as "0")**	**70%**		**60%**		**0%**	**43.3%**
TOTAL (%)	**(Considering** " unprocessed photographs " **as "missing value")**	**70%**		**75%**		**0%**	**61.9%**
