# Supplementary material for: Accuracy of AI Tools in the Diagnosis of Benign, Potentially Malignant and Malignant Oral Lesions: A Pilot Study
Source: J Clin Med. 2026 Mar 30;15(7):2638. doi: 10.3390/jcm15072638 (PMC13072891; doi:10.3390/jcm15072638)
Supplement: Supplementary file 1 [file jcm-15-02638-s001.zip › Supplemental Table S5.pdf]

Supplemental Table S5 - Diagnostic Accuracy metrics analysis obtained from TP, FN, TN, FP results

Metric	Scenario (unprocessed photos)	ChatGPT % (95% CI)	Gemini % (95% CI)	Copilot % (95% CI)	Global p-value*	ChatGPT vs Gemini**	ChatGPT vs Copilot**	Gemini vs Copilot**
**Sensitivity**	as "0"	70.00 [39.7–89.2]	60.00 [31.3–83.2]	0.00 [0.0–25.9]	0.003	0.751	0.003	0.011
	as "missing"	70.00 [39.7–89.2]	75.00 [40.9–92.9]	0.00 [0.0–39.0]	0.057	1.000	0.070	0.061
**Specificity**	as "0"	65.00 [43.3–81.9]	75.00 [53.0–88.6]	35.00 [18.1–56.7]	0.028	0.731	0.113	0.025
	as "missing"	65.00 [43.3–81.9]	78.95 [56.7–91.5]	46.67 [23.2–71.5]	0.147	0.480	0.321	0.075
**Accuracy**	as "0"	66.67 [48.8–80.8]	70.00 [52.1–83.3]	23.33 [11.5–41.2]	<0.001	1.000	0.002	0.001
	as "missing"	66.67 [48.8–80.8]	77.78 [59.2–89.4]	38.89 [20.3–61.4]	0.027	0.391	0.077	0.013
**PPV**	as "0"	50.00 [28.0–72.0]	54.55 [28.0–78.7]	0.00 [0.0–39.0]	0.006	1.000	0.006	0.003
	as "missing"	50.00 [28.0–72.0]	60.00 [31.3–83.2]	0.00 [0.0–48.9]	0.023	0.697	0.022	0.013
**NPV**	as "0"	81.25 [57.0–93.4]	78.95 [56.7–91.5]	41.18 [21.6–64.1]	0.020	1.000	0.032	0.039
	as "missing"	81.25 [57.0–93.4]	88.24 [65.7–96.7]	70.00 [39.7–89.2]	0.501	0.656	0.644	0.326

Legend: TP - true positive; FP - false positive; TN - true negative; FN - false negative; CI – confidence interval; PPV: Positive Predictive Value; NPV: Negative Predictive Value; * For Global p-value Pearson's Chi-Square (or Fisher's Exact Test for counts < 5) was used; ** for pairwise comparisons a Fisher's Exact Test for head-to-head model differences was used.

Accuracy of AI Tools in the Diagnosis of Benign, Potentially Malignant and Malignant Oral Lesions: a pilot study
